# Neural mechanisms of goal-directed behavior: outcome-based response selection is associated with increased functional coupling of the angular gyrus

**DOI:** 10.3389/fnhum.2015.00180

**Published:** 2015-04-10

**Authors:** Katharina Zwosta, Hannes Ruge, Uta Wolfensteller

**Affiliations:** Department of Psychology, Technische Universität DresdenDresden, Germany

**Keywords:** goal-directed behavior, action control, angular gyrus, fMRI, functional connectivity

## Abstract

Goal-directed behavior is based on representations of contingencies between a certain situation (S), a certain (re)action (R) and a certain outcome (O). These S-R-O representations enable flexible response selection in different situations according to the currently pursued goal. Importantly however, the successful formation of such representations is a necessary but not sufficient precondition for goal-directed behavior which additionally requires the actual usage of the contingency information for action control. The present fMRI study aimed at identifying the neural basis of each of these two aspects: representing vs. explicitly using experienced S-R-O contingencies. To this end, we created three experimental conditions: S-R-O contingency present and used for outcome-based response selection, S-R-O contingency present but not used, and S-R-O contingency absent. The comparison between conditions with and without S-R-O contingency revealed that the angular gyrus is relevant for representing S-R-O contingencies. The explicit usage of learnt S-R-O representations in turn was associated with increased functional coupling between angular gyrus and several subcortical (hippocampus, caudate head), prefrontal (lateral orbitofrontal cortex (OFC), rostrolateral prefrontal cortex (RLPFC)) and cerebellar areas, which we suggest represent different explicit and implicit processes of goal-directed action control. Hence, we ascribe a central role to the angular gyrus in associating actions to their sensory outcomes which is used to guide behavior through coupling of the angular gyrus with multiple areas related to different aspects of action control.

## Introduction

Goal-directed behavior is characterized by choosing actions according to the outcomes they produce in a certain situation. Thus, the prerequisite of being able to act in a goal-directed manner is to have acquired representations of response-outcome relationships in a certain situation (S-R-O representations), and hence being able to anticipate the future outcome of an action. However, this representation of an S-R-O relationship alone does not make behavior goal-directed as, in addition, these representations have to be actually used in order to select the response (see also de Wit and Dickinson, 2009). Evidence for this dissociation of both processes reach back to classic latent learning experiments with rats (Tolman and Honzik, 1930) demonstrating that S-R-O representations can be acquired without being used to guide behavior as long as the outcome is not motivationally relevant. More recent behavioral experiments in humans also demonstrated that S-R-O representations are built while their explicit implementation additionally depends on a goal-directed action control mode (Herwig et al., 2007; Pfister et al., 2011; Zwosta et al., 2013).

Being able to adjust behavior to the desired consequences in a certain situation is a core ability of flexible behavior that is assumed to be impaired in several psychological disorders such as e.g., obsessive-compulsory disorder (Gillan et al., 2011) and addiction (Everitt et al., 2008). Yet, in spite of its behavioral relevance little is known about the neural basis that enables people to flexibly adjust their behavior to outcomes they want to reach. In particular, the mere representation of S-R-O contingencies and their explicit usage have not been systematically disentangled. However, several candidate brain regions can be identified from previous neuroimaging studies that have investigated goal-directed action both in the ideomotor framework and in the instrumental conditioning framework (for reviews, see e.g., Wolfensteller and Ruge, 2012; Dolan and Dayan, 2013). While ideomotor learning accounts investigate goal-directed behavior in terms of sensory non-incentive outcome anticipations (James, 1890; Greenwald, 1970), instrumental learning accounts focus on the dependence of actions on incentive outcomes (reward and punishment). Previous research on ideomotor processes primarily focused on the passive retrieval of action-outcome associations. Subjects first engaged in a learning phase during which they contingently experienced that specific responses were followed by specific (mostly auditory) outcomes. In a subsequent test phase they were presented with outcomes that were previously coupled to a certain response or did not appear in the learning phase. Brain activation associated to the processing of action outcomes was found to be higher in the supplementary motor area (SMA) and hippocampus (Elsner et al., 2002) as well as angular gyrus and cerebellum (Melcher et al., 2008).

Recently two fMRI studies investigated BOLD activation dynamics during the learning of contingent vs. non-contingent action outcomes, both again suggesting an important role of the angular gyrus. Consistent with “passive effect priming” studies Melcher et al. (2013) found a stronger decrease of activation across an extended learning phase in the hippocampus and angular gyrus when effects were contingent on responses. Angular gyrus activity during learning also predicted the hippocampal response to outcome stimuli in the test phase, suggesting that the angular gyrus is involved both in associating responses and outcomes and in retrieving the response in face of the outcome. In another recent study, the angular gyrus (and cerebellum) were also found to be involved in correct response selection when actions could be chosen according to specific outcomes, emphasizing the role of this area in the selection of responses that aim at specific outcomes (Noonan et al., 2011). The specific role of the angular gyrus in intentional action selection is further corroborated by the finding that electrical stimulation of the this area led patients to report motor intentions without actually performing a movement (Desmurget et al., 2009).

In a study conducted by Noonan et al. (2011) another area whose activation was associated to correct response selection of actions leading to specific outcomes was the anterior cingulate cortex (ACC). Part of this area, the rostral cingulate zone (RCZ) was also found to be involved in freely chosen actions (compared to forced choices) (Lau et al., 2004; Mueller et al., 2007) which suggests an involvement in intentional action selection.

Besides these areas, instrumental conditioning and ideomotor learning studies consistently suggest the caudate nucleus as another important brain region for the learning of R-O associations (Tricomi et al., 2004; Melcher et al., 2013). Specifically, instrumental conditioning studies suggest that the caudate nucleus (i.e., dorsomedial striatum) is more involved in instrumental behavior and the putamen (i.e., dorsolateral striatum) more in habitual behavior (Yin and Knowlton, 2006; Balleine et al., 2009; Tricomi et al., 2009). Similarly, during ideomotor learning, Ruge and Wolfensteller (2013) showed an involvement of dorsomedial striatum (caudate head) during the rapid short timescale acquisition of S-R-O associations together with ventromedial striatum (caudate head and nucleus accumbens) and orbitofrontal cortex (OFC) areas.

The aim of the present study was to systematically investigate the involvement of these areas in representing S-R-O contingencies on the one hand and in actively using them in the course of goal-directed response selection on the other hand. To this end, we compared two conditions with a hierarchical relationship between stimuli, responses and outcomes, where R-O contingencies in one stimulus context were inverted in the other stimulus context, to a control condition with random outcomes in order to determine activation related to the representation of S-R-O associations. Additionally, we manipulated the active usage of these S-R-O representations by instructing participants either to choose their responses based on outcomes (hence rendering the outcome motivationally relevant) or based on stimulus-response rules (with no reference to the outcome).

## Materials and Methods

### Participants

Thirty-three right-handed neurologically and psychiatrically healthy subjects with normal color vision participated (19 female, mean age: 23.8, SD: 2.9). The experimental protocol was approved by the Ethics Committee of the Technische Universität Dresden. All participants gave written informed consent prior to taking part in the experiment and were paid 8€ per hour for their participation.

### Experimental Paradigm

We compared three different experimental conditions which were manipulated within-subject. An outcome-based condition which included both S-R-O representations as well as their usage, a stimulus-based condition where S-R-O representations were present but not used, and finally a control condition with random outcomes. By comparing outcome-based and stimulus-based conditions to a control condition with random outcomes we could identify brain activation related to S-R-O representation while comparing the outcome-based with the stimulus-based condition reflected the active usage of S-R-O contingencies. For an overview of the conditions, see Figure 3.

#### Manipulation of S-R-O Contingency

**Figure 1:**
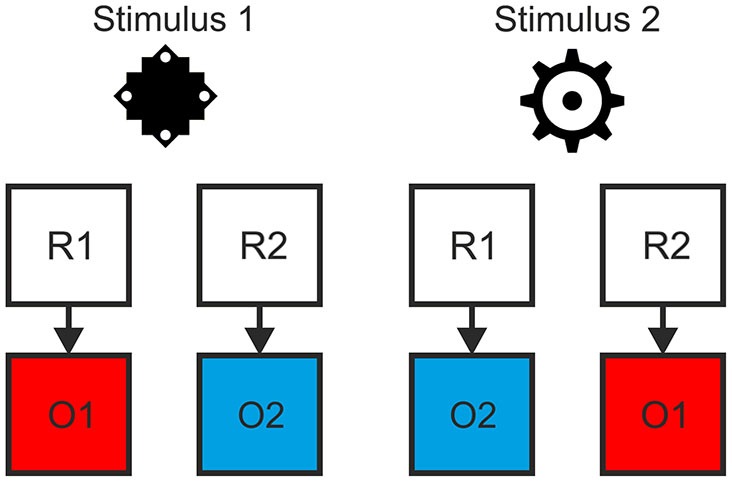
**Example of the hierarchical relationship between stimuli, responses and outcomes**. The contingency between response and outcome in situation 1 (as indicated by stimulus 1) was inverted in situation 2 (stimulus 2).

We employed a task with a hierarchical relationship between stimuli, responses and specific outcomes, such that R-O contingencies in one context were inverted in the other context (see Figure 1). Responding to stimulus 1 by pressing the left key (R1) led to color A (O1) while pressing the right key (R2) led to color B (O2). For stimulus 2 this was inverted: Pressing the left key (R1) led to color B (O2), pressing the right key (R2) to color A (O1). This was the case for both the outcome-based and the stimulus-based condition. Hence, there was a perfect contingency between a response in a situation and the resulting outcome in both conditions. At the same time, there was no systematic relationship between stimuli and responses or stimuli and outcomes. Thereby we could avoid confounding S-R-O contingency with S-R or S-O contingencies. In a control condition outcomes were delivered randomly. Here, pressing a specific key in response to specific stimulus could lead to two different outcomes with the same probability and hence no S-R-O contingency representation could be formed.

#### Manipulation of Outcome-Based Response Selection

The second manipulation referred to the way the response was chosen. As each stimulus implied two response options (cf. Figure 1), additional information was required to resolve these ambiguous S-R rules. Crucially, S-R rule ambiguity was resolved differently based on two different types of cues preceding the stimulus. In the outcome-based condition the cues were colored frames signaling which future color outcome was to be produced by responding to the stimuli. This promoted the usage of the acquired S-R-O representations. By contrast, in the stimulus-based condition and in the control condition the cues were different frame patterns that indicated which S-R rule to apply in the current trial and made no reference to the outcome. Hence, in the outcome-based condition response selection was controlled by a rule emphasizing the desired outcome, while in both the stimulus-based condition and the control condition response selection was controlled by a rule emphasizing the stimulus.

**Figure 2:**
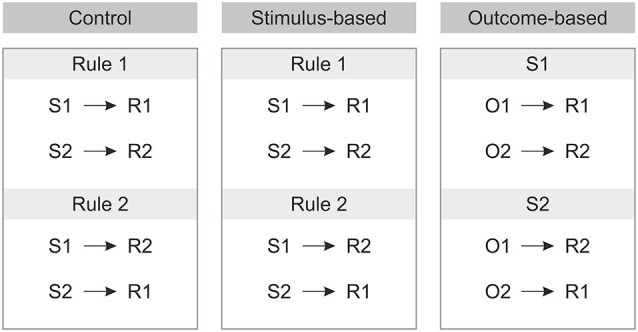
**Instructions for each of the three conditions prior to the experiment**. S: Stimulus, R: Response, O: Outcome.

Prior to the experiment the tasks were instructed either in terms of S-O-R relationships—in the outcome-based condition (In the presence of stimulus 1, to get O1 perform R1 and to get O2 perform R2; In the presence of stimulus 2 to get O1 perform R2 and to get O2 perform R1), or in terms of S-R rules—in the stimulus-based and control conditions (Rule 1: If stimulus 1 appears, perform R1, if stimulus 2 appears perform R2; Rule 2: If stimulus 1 appears press R2, if stimulus 2 appears press R1, see Figure 2).

In previous behavioral studies we have addressed the question whether this paradigm indeed renders only actions in the outcome-based but not in the stimulus-based condition controlled by the outcome (Zwosta et al., 2013). In these experiments we used the same paradigm except that the outcome location could be either compatible or incompatible to the response location. Differences in response times for spatially compatible and incompatible trials indicate that the outcome (appearing after the response) must have been anticipated during response selection and hence, response selection was guided by the anticipated outcome. This effect was observed only for the outcome-based condition, but not for both stimulus-based and control conditions indicating that only the outcome-based instruction leads to outcome anticipation during response selection and thus a goal-directed action control mode. Hence, we could make sure that actions are controlled by their outcomes only in the outcome-based condition. Furthermore, we could demonstrate that the action control mode was not transferred from one condition block to the next. We did not include this manipulation of spatial R-O compatibility in the present fMRI study because otherwise neural correlates of response-outcome interference and outcome anticipation would have been confounded.

#### Trial Procedure

**Figure 3:**
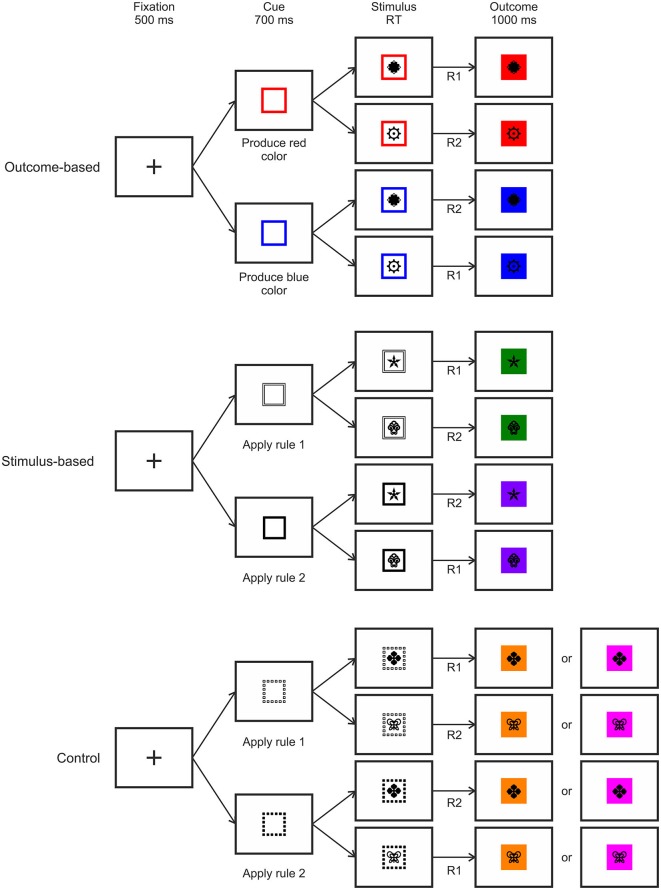
**Examples of trial procedures with correct responses for the three experimental conditions**. RT: Response time, R: Response.

The trial procedure was identical for all three conditions except for cue type and outcome contingency (see Figure 3). Each trial started with a fixation cross displayed at the center of the screen for 500 ms. This was replaced by the cue frame presented for 700 ms which indicated which one of the two outcomes should be produced (in the outcome-based condition) or which one of the two S-R rules should be applied (in the stimulus-based and the control conditions). Afterwards, the stimulus appeared in the center of the cue frame for maximally 2000 ms or until a response was made. If the response was correct then the background of the stimulus was colored, and stimulus and color stayed on-screen for 1000 ms. Importantly, in outcome- and stimulus-based conditions, the color was chosen according to the respective hierarchical S-R-O scheme while in the control condition one of two colors was chosen at random. If the response was incorrect then the word “wrong” appeared in the center and the background turned to the alternative color, thus keeping the S-R-O contingency intact. If no response was made then “too slow” was displayed leading to no outcome color.

#### Stimuli

For each condition a unique pair of stimuli and outcome colors was used. These three pairs of black-and-white stimulus pictures as well as three pairs of outcome colors were randomly assigned to the three conditions. All stimuli were taken from the Creative Symbol Collection of Matton Images. Responses were made with the index fingers of the left and the right hand. Cues for the outcome-based condition consisted of colored square frames that indicated the outcome color that was to be produced. For the conditions with stimulus-based instructions the cues consisted of black or white square frames that indicated which rule to apply. For one of the two conditions the lines of the square frames were solid while for the other one they were dashed.

### Experimental Procedure

The experiment consisted of a practice phase outside of the scanner in order to make subjects familiar with the tasks and ensure a sufficiently low error rate. This was then followed by the main fMRI experiment. The three conditions alternated within a block design which provides optimal statistical power (Friston et al., 1999), and hence the possibility to detect subtle differences between the conditions, and is well-suited to investigate differences in functional connectivity (Gitelman et al., 2003).

#### Pre-Experimental Instruction and Practice

During the practice phase subjects were seated in a separate room and learned the conditions in separate blocks. The order of the conditions was balanced across participants.

In the outcome-based condition, the two-step instruction procedure started with a display showing the first stimulus and the two outcome colors that would be produced by pressing the left and right key respectively. This was followed by eight trials including only the first stimulus. Afterwards, the same procedure was repeated for the second stimulus, which was followed by 32 intermixed trials containing either one of the stimuli. For the stimulus-based and the control conditions, the two-step instruction procedure started with a display showing the two stimuli and which response to make after them when S-R rule 1 was to be applied. Accordingly, after eight practice trials the second S-R rule was instructed. Again, thereafter, 32 intermixed trials containing either one of the two rules were presented. When all three conditions were instructed following this procedure, the final practice phase started which matched the proper experiment. It comprised three blocks of 16 trials for each condition, which were separated by a display saying “the next block is about to start” for 3000 ms. During practice, error trials were repeated until a correct response was made. Otherwise, trial timing was identical to the main fMRI experiment.

#### fMRI Experiment

The main fMRI experiment started with a display showing a summary of the instructions for each condition. After this there were three runs each containing nine task blocks with 16 trials. Between the task blocks there was a 30 s baseline during which a fixation cross was presented. After 29 s the cross shortly disappeared for 500 ms indicating that the next block was about to start. The order of the condition blocks was randomized with the constraint that each run started with another condition and that transition frequencies were balanced. A unique randomization was used for each participant. One block lasted about 45 s depending on the individual response speed.

### Imaging Procedure

MRI data were acquired on a Siemens 3T whole body Trio System (Erlangen, Germany) with a 16 channel head coil. Ear plugs dampened scanner noise. Before the experiment started, structural images were acquired using a T1-weighted sequence (TR = 1900 ms, TE = 2.26 ms, TI = 900 ms, flip = 9°) with a resolution of 1 mm × 1 mm × 1 mm. Functional images were acquired using a gradient echo planar sequence (TR = 2000 ms, TE = 30 ms, flip angle = 80°). Each volume contained 32 slices that were measured in ascending order. The voxel size was 4 mm × 4 mm × 4 mm (gap: 20%). The experiment was controlled by E-Prime 2.0.

### Data Analysis

The fMRI data were analyzed using SPM8 based on MATLAB 7.14. First, the functional images were spatially realigned and unwarped. Each participant’s structural image was co-registered to the mean functional image and segmented. Spatial normalization to MNI space was performed using DARTEL with a spatial resolution of 3 mm × 3 mm × 3 mm. Images were then smoothed with a Gaussian Kernel of 8 mm full-width at half maximum.

#### Standard Univariate Analysis

Data analysis was performed using the General Linear Model (GLM) approach implemented in SPM8. We modeled each condition block (approximately 45 s, depending on the individual response speed) by a boxcar function convolved with SPM canonical HRF. We included one regressor per condition and run which amounted to nine regressors (three conditions in three runs). Each run contained three blocks of each condition and the baseline was modeled implicitly. The high pass filter was set to 1/180 Hz. For each subject contrast images were generated for all pairs of the three conditions. Differences between the conditions were then analyzed on the group level using one-sample *t*-tests. In addition to the whole brain analysis, we conducted ROI analyses for the angular gyrus, the caudate nucleus, the hippocampus, the OFC, the cerebellum, the SMA and the ACC based on previous results (Elsner et al., 2002; Tricomi et al., 2004, 2009; Yin and Knowlton, 2006; Melcher et al., 2008, 2013; Balleine et al., 2009; Desmurget et al., 2009; Noonan et al., 2011; Ruge and Wolfensteller, 2013). All anatomical ROIs were derived from the automatic anatomic labeling atlas (AAL). Correction for multiple comparisons was done by determining the number of voxels necessary to meet a cluster-corrected threshold of *p* < 0.05 through AfNi’s Monte Carlo Simulations (3dClustSim) with the initial voxel-wise threshold set to *p* < 0.001.

#### PPI Analysis

In order to assess differences in functional connectivity between the conditions we conducted a psycho-physiological interaction (PPI) analysis as implemented in SPM8, using a sphere of 6 mm around the peak voxel coordinate in the right angular gyrus that showed stronger activation in outcome-based and stimulus-based blocks compared to the control condition (MNI seed coordinates: 45, −57, 33). Differences in functional connectivity were again computed for the contrasts between all pairs of conditions. Significance was set to cluster-corrected *p* < 0.05 based on Monte Carlo simulations. Additionally, we performed an exploratory analysis reporting clusters with a more lenient cluster size of 20 voxels with the voxel-based threshold set to *p* < 0.001.

## Results

### Behavioral Results

**Figure 4:**
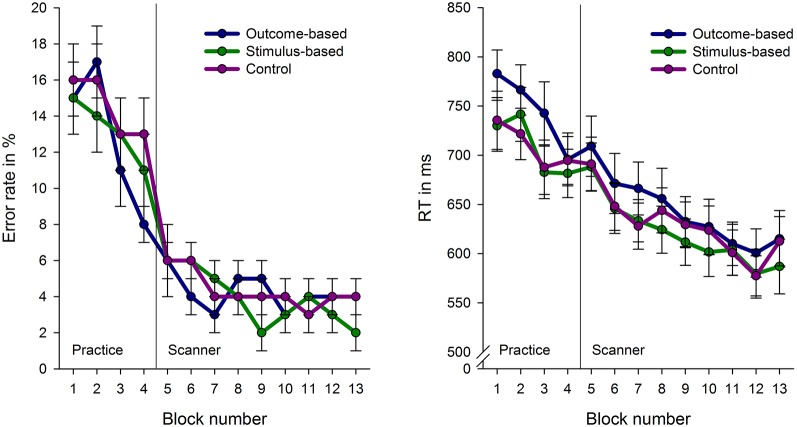
**Error rates and response times (RT) for the experimental conditions across the course of the experiment**. Each block comprises 16 trials with the exception of block 1 which consist of 32 trials. Error bars indicate standard errors.

Figure 4 depicts error rates and response times for the conditions in the course of the experiment. Response times and error rates were analyzed separately for the practice phase and fMRI experiment using a repeated measures ANOVA with condition as a within-subject factor.

#### Practice

Due to technical problems behavioral data of the practice phase was lost for two subjects. Analyses revealed no differences in error rates between the conditions, *F* < 1. However, RTs were influenced by the experimental conditions, *F*_(2,64)_ = 6.82, *p* = 0.001. Subjects’ responses were slower in the outcome-based condition compared to the stimulus-based and control conditions, *t*_(30)_ = 2.70, *p* = 0.011 and *t*_(30)_ = 3.18, *p* = 0.003, respectively. RTs between the stimulus-based and the control conditions did not differ, *t* < 1, which indicates that subjects did neither benefit nor suffer from contingent outcomes in the stimulus-based condition.

#### fMRI Experiment

The practice-related behavioral differences were no longer present during the fMRI experiment: There were no significant differences between the three experimental conditions in either RT or error rate (*F*s < 1). Participants were able to perform the tasks in an accurate and timely manner: Mean error rate was 4.2% (SE: 0.6%, range 0.5–11.6%) and mean RT in correct trials was 613 ms (SE: 19 ms). Accordingly, brain activation differences cannot be attributed to performance level differences between the conditions.

### Imaging Results

#### Standard Univariate Analysis

**Figure 5:**
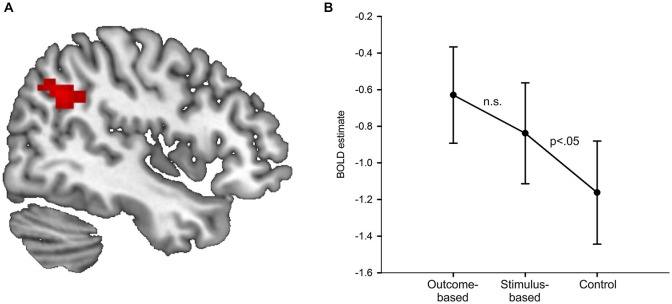
**Results of the univariate analysis. (A)** Result of the comparison of brain activation between the outcome-based and the control condition (cluster-corrected *p* < 0.05 corresponding to at least 66 adjacent voxels with uncorrected *p* < 0.001). Activation in the right angular gyrus was significantly higher during outcome-based blocks. **(B)** BOLD estimates for each of the conditions within the angular gyrus ROIs derived from a leave-one-subject-out analysis. Error bars reflect standard errors.

Results of the univariate analysis are summarized in Table 1. Whole brain analysis revealed significantly stronger activation in outcome-based blocks compared to control blocks only in the right angular gyrus (see Figure 5A), as well as in the medial occipital cortex. Additional ROI analyses revealed stronger activity only in the left angular gyrus but in no other pre-defined ROI. None of the other contrasts, including the comparison between outcome-based and stimulus-based conditions, revealed significant differences in activation.

**Table 1:** **Summary of the results of the univariate analysis**.

Region	MNI Coordinates			*T*_max_	Cluster size
**Outcome-based > Control**
R Angular gyrus	45	−57	33	4.58^1^	69
L Angular gyrus	−57	−63	24	3.49^2^	9
R Occipital lobe	9	−81	24	4.05^1^	87

*No other contrast yielded any significant results*.

*^1^Whole-brain cluster-corrected for multiple comparisons p < 0.05, ^2^Cluster-corrected for multiple comparisons adjusted for the size of the ROI p < 0.05*.

In order to determine whether the activation difference we found in the specific part of the angular gyrus between the outcome-based and the control condition was due to mere representation or actual usage of S-R-O contingencies, we computed two directed pair-wise comparisons on the condition-specific activation estimates (stimulus-based > control, stimulus-based < outcome-based). If angular gyrus activity was related to the presence of an S-R-O representation, then it should be higher in the stimulus-based condition than the control condition but should not differ between the outcome-based and the stimulus-based condition. If, instead, angular gyrus activity was primarily driven by the usage of S-R-O representations, activity in the stimulus-based condition should be similar to the control condition and smaller than in the outcome-based condition.

In order to ensure that ROI definition was independent of value extraction, and thus to avoid circular analysis (Kriegeskorte et al., 2009) we applied the leave-one-subject-out method (Esterman et al., 2010). Activation values for each subject were extracted from a ROI derived from data from the rest of the subjects. Thus, for each subject we calculated the group-based contrast between the outcome-based and the control condition without this subject. Then we determined the largest cluster with its peak voxel within the anatomical boundaries of the right angular gyrus at a voxel-based threshold of *p* < 0.001. Notably, for each of the 33 ROIs the peak voxel was always identical to the peak voxel in the whole-group analysis while cluster sizes ranged from 34 to 91 voxels (mean: 57 voxels). Finally, for the remaining subject we extracted the mean activation values of the voxels within this cluster for each of the three conditions compared to baseline.

Figure 5B shows the resulting activation estimates separately for each condition as compared to the implicit fixation baseline. The pair-wise comparisons of interest showed that activation in the stimulus-based condition was significantly higher than in the control condition *t*_(32)_ = 1.78, *p* = 0.042 (Bonferroni-corrected for two comparisons, one-sided). The difference between outcome-based and stimulus-based conditions was not significant, *t*_(32)_ = 1.10, *p* = 0.139 (Bonferroni-corrected for two comparisons, one-sided). This suggests that angular gyrus activity is related to S-R-O representation. Of note, the activation estimates for the stimulus-based condition were numerically in between the outcome-based and the control condition. While the significant difference between stimulus-based and control condition clearly demonstrates that the mere representation of S-R-O contingencies is sufficient to elicit enhanced activity in the angular gyrus, the non-significant difference between stimulus-based and outcome-based conditions calls for a more cautious interpretation. While in the present sample of *N* = 33, the usage of these representations did not additionally boost angular gyrus activation, such an arguably more subtle increase might be detected with more statistical power.

#### PPI Results

Results of the PPI analysis are summarized in Table 2. Condition-dependent differences in functional connectivity of the right angular gyrus were found for the comparisons between outcome-based and stimulus-based as well as between outcome-based and control conditions. Specifically, in the outcome-based condition compared to both other conditions, an increased functional connectivity between the right angular gyrus and specific cortical and subcortical areas was revealed. All other comparisons between conditions did not yield any reliable results (less than 10 contiguous voxels with *p* < 0.001).

**Table 2:** **Summary of the PPI results**.

Region	MNI Coordinates			*T*_max_	Cluster size
**Outcome-based > Stimulus-based**
R Hippocampus	21	−33	0	4.94^1^	72
L Hippocampus	−21	−33	3	4.37^1^	60
R Caudate head	6	15	3	3.79^2^	12
L Caudate head	−6	12	9	4.09^2^	10
R Lateral OFC	36	24	−18	4.13^2^	30
R Lateral OFC	48	27	−6	3.87^2^	11
R Occipital lobe	30	−90	−6	4.16^1^	117
R Occipital lobe	18	−66	24	4.48^3^	23
Cerebellum	−30	−78	51	4.55^2^	46
Cerebellum	0	−48	−36	4.49^2^	28
L RLPFC	−36	54	12	4.38^3^	44
L SPL	−21	−66	48	3.95^3^	42
**Outcome-based > Control**
R Caudate head	6	15	6	4.93^2^	15
L Caudate head	−15	18	0	3.66^2^	6
R Occipital lobe	30	−93	12	5.26^1^	100
Cerebellum	3	−54	−9	4.05^2^	24
L Occipital lobe	−9	−102	0	4.12^3^	49
(L RLPFC)	−39	57	12	(3.77)	8
**Stimulus-based > Control**: no significant results (overall 8 voxels with p < 0.001, uncorrected).

*^1^Whole-brain cluster-corrected for multiple comparisons p < 0.05, ^2^Cluster-corrected for multiple comparisons adjusted for the size of the ROI p < 0.05, ^3^more than 20 adjacent voxels with uncorrected p < 0.001*.

##### Outcome-based condition > stimulus-based condition

**Figure 6:**
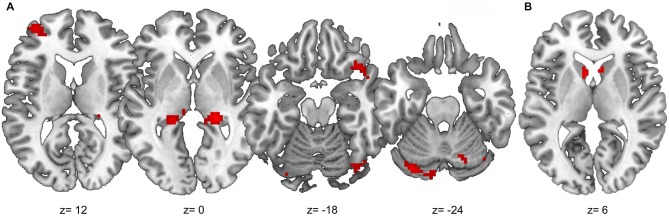
**Results of the PPI analysis with stronger functional connectivity to the right angular gyrus in the outcome-based condition compared to the stimulus-based condition. (A)** Clusters with at least 20 adjacent voxels with uncorrected *p* < 0.001. **(B)** Anatomical caudate ROI, voxels with uncorrected *p* < 0.001.

The results are shown in Figure 6. Whole-brain analysis resulted in stronger connectivity between the right angular gyrus and clusters in both hippocampi and the right occipital lobe. Moreover, ROI analyses revealed stronger functional connectivity between the right angular gyrus and the caudate head bilaterally, right lateral OFC (two adjacent clusters) and cerebellum. The exploratory analysis, based on at least 20 adjacent voxels with *p* < 0.001, furthermore yielded a cluster in the left rostrolateral prefrontal cortex (RLPFC) and a cluster in the superior parietal lobe (SPL) as well as an additional cluster in the right occipital lobe. However, there were no voxels in the SMA or ACC with an uncorrected *p* < 0.001.

##### Outcome-based condition > control condition

Similar results were found for the comparison between the outcome-based and control condition (see Table 2). Whole-brain analysis yielded significantly stronger connectivity to the right occipital lobe. ROI analyses identified clusters in bilateral caudate head and the cerebellum. Again, there was a tendency towards stronger activation in the left RLPFC which did however, not reach corrected significance (*T*_max_ = 3.89, 8 adjacent voxels with *p* < 0.001).

Together, the increased functional connectivity of the angular gyrus in the outcome-based condition compared to both the stimulus-based and the control conditions and the absence of a difference in functional connectivity between the latter two conditions indicates that these functional couplings reflect neural mechanisms related to outcome-based response selection.

## Discussion

The aim of the present study was to investigate the neural basis of two components of goal-directed behavior: the representation of S-R-O contingencies and their actual usage in outcome-based response selection, i.e., the choice of actions according to their anticipated outcomes. To this end we compared an outcome-based condition where people were instructed to produce a specific outcome with a stimulus-based condition where people were instructed to respond according to stimulus-response rules and a control condition with random outcomes.

We found that if outcomes were contingent on responses to stimuli (i.e., in the outcome-based and stimulus-based condition), activity in the right angular gyrus was stronger than in the control condition with random action outcomes. Furthermore, the actual integration of outcomes into response planning, revealed by comparing outcome-based and stimulus-based conditions, was accompanied by increased functional connectivity between the angular gyrus and hippocampus, caudate head, lateral OFC, cerebellum and, at a more lenient threshold, also RLPFC.

Hence, the theoretical and empirical notion that, while the representation of stimulus-response-outcome contingencies is a prerequisite of goal-directed behavior, these associations are not automatically used to guide behavior (Pfister et al., 2010; Zwosta et al., 2013) is supported on the neural level. Our results suggest that these processes are reflected in a stronger involvement of the angular gyrus when outcomes are predictable independent of their usage, while outcome-directed response selection is additionally associated with stronger functional connectivity to several brain areas involved in goal-directed action control.

Note that the aim of this study was not to contrast goal-directed and habitual behavior but to isolate two distinct aspects of goal-directed behavior. Hence, all conditions can be considered goal-directed to some degree, as participants were motivated to press the correct response and to avoid errors. Furthermore, as the conditions did not differ in the number of repetitions of cue-stimulus-response pairings, we would not expect differences in habitualization. Instead, the critical difference between the outcome-based and the stimulus-based condition was the explicit integration of the (non-incentive) specific outcome into response selection.

### Representation of S-R-O Associations

A first main finding of the present study is that the angular gyrus plays a central role in relating sensory outcomes to actions in a certain situation. Activity in the angular gyrus was higher whenever specific action outcomes were predictable. This is consistent with its previous characterization as an integration zone of bottom-up and top-down processes which is involved in a multitude of cognitive functions (Seghier, 2013). In the context of action outcomes, it was previously argued that the angular gyrus plays a central role in predictive coding by comparing intended to actual action outcomes: It was found to be more strongly activated when predictions are not met, thus signaling expectancy-incongruent events (Sirigu et al., 2004; Farrer et al., 2008; Spengler et al., 2009; Liljeholm et al., 2011; Seghier, 2013). In contrast, we find that the angular gyrus is more strongly involved when expectancies about outcomes exist, even if these are always fulfilled, compared to a control condition with unpredictable outcomes. Hence, we suggest that the angular gyrus is generally involved when predictions can be made about the outcomes of actions, by representing action-outcome contingencies, and not only when these predictions are violated.

### Outcome-Based Response Selection

Our results further suggest that outcome-based action control is relying on the functional coupling of the angular gyrus with other prefrontal, subcortical and cerebellar areas. Hence, while the conditions did not differ regarding activity in either of the goal-directed brain areas, functional connectivity of the angular gyrus with the goal-directed network was enhanced in the outcome-based condition.

The notion that outcome-based response selection is not reflected in activation differences but instead in differences in functional connectivity is in line with previous findings on initial learning processes of action contingent outcomes (Ruge and Wolfensteller, 2013), instrumental trial and error learning processes (Li et al., 2011), and reward processing (Camara et al., 2009) and seems to be a rather general feature of large-scale functional integration in the brain (Cross and Iacoboni, 2013).

The involvement of the angular gyrus in intentional actions is corroborated by findings that relate lesions in this particular area with deficits in awareness of intention (Sirigu et al., 2004) as well as a study demonstrating that electrical stimulation of the angular gyrus leads to patients reporting motor intentions (Desmurget et al., 2009). Our results further specify the role of this brain structure in goal-directed behavior by showing that its true importance is related to the actual usage of learnt stimulus-response-outcome representations and emerges via large-scale functional couplings with specific brain areas, notably hippocampus, caudate head, lateral OFC, cerebellum and RLPFC. Some suggestions for the possible functions of these couplings are made in the following paragraphs. Of course in using a blocked design that is well suited to investigate functional connectivity, we can only speculate at which phase of a trial the reported differences occurred. The attribution of the results to temporal subprocesses is an important task of itself that should definitely be addressed in future studies.

Previous studies have already suggested an involvement of the hippocampus in learning and retrieval of action-outcome relationships (Melcher et al., 2008, 2013). Moreover, successful recollection of episodic memory content engages a hippocampal-parietal memory network as revealed by resting state connectivity analysis (Vincent et al., 2006). Given that the angular gyrus is involved in the representation of stimulus-response-outcome contingencies, functional connectivity to the hippocampus might reflect the explicit retrieval of these contingencies in order to select the appropriate response to produce the desired outcome.

The functional connectivity between angular gyrus and caudate head might reflect a more implicit part of outcome-based response selection. The anterior caudate was found to be more active during early instrumental behavior, but decreased as the action became more habitual, i.e., independent of the outcome (Yin and Knowlton, 2006; Balleine et al., 2009).

Another area that is typically found to be involved in goal-directed behavior is the OFC. In particular, the lateral OFC was previously found to be involved in associating a specific (valued) outcome with a stimulus (Walton et al., 2010; Rushworth et al., 2011; Noonan et al., 2012). In case of outcome-based response planning this might reflect the assignment of a value to the desired outcome, thereby choosing the goal that is to be pursued and enabling switching between the different desired outcomes.

Furthermore, we found increased coupling of the angular gyrus to the cerebellum. Just as the angular gyrus, also the cerebellum is associated with predictive coding (Blakemore et al., 2001). However, while the angular gyrus was associated with conscious prediction of action consequences, cerebellum was suggested to be involved in the implicit prediction of sensory events resulting from actions (Blakemore and Sirigu, 2003; Sirigu et al., 2004). The observed stronger connectivity between angular gyrus and cerebellum in the outcome-based condition might thus indicate a stronger guidance of anticipatory cerebellar processes by conscious action-outcome expectation processes supported by the angular gyrus.

Additionally, there was a tendency towards stronger functional connectivity to left rostrolateral PFC (RLPFC). While this area is not part of the typical brain network engaged in goal-directed behavior, it is usually involved when multiple operations have to be coordinated (Ramnani and Owen, 2004), in prospective memory tasks as well as when an alternative task set is kept in mind during the performance of the ongoing task (Koechlin and Hyafil, 2007). As the present experiment required the maintenance of two S-R rules in order to elicit one outcome, we speculate that RLPFC engagement could reflect coordination of those simultaneous rules (see also Wolfensteller and von Cramon, 2010). Hence, one suggestion would be that coupling between angular gyrus and RLPFC reflects the hierarchical contextualization of response-outcome associations in the present experiment.

Finally, in studies investigating intentional goal-directed behavior by comparing free-choice to forced-choice actions, the former are usually found to lead to stronger activation in the pre-SMA and dorsal ACC/RZC (e.g., Lau et al., 2004; Mueller et al., 2007) which led to the assumption that the medial frontal cortex is central to intentional action control. Free-choice actions are, however, confounded with conflict resolution as they require a choice between multiple competing response options (Brass and Haggard, 2008). Using only forced-choice tasks we did not find any involvement of pre-SMA or ACC even at a lenient threshold. However, Noonan et al. (2011) found that ACC activity correlated with the probability of correct response selection when actions were followed by specific outcomes which implies an involvement in outcome-based response selection. A possible explanation for why we do not find ACC involvement neither in the univariate analysis nor in the PPI analysis might be that we investigated outcome-based response selection after S-R rules were explicitly instructed and participants had practiced them. In contrast, in the study conducted by Noonan et al. (2011) participants were still acquiring S-R rules, and interestingly, the correlation between ACC activity and performance substantially diminishes when higher performance levels matching the ones in our study are reached. Together, this might indicate that ACC is primarily involved when there is still a certain ambiguity about which response leads to the desired outcome. Hence, while keeping in mind that null results can only be interpreted with caution we tentatively suggest that the ACC and pre-SMA might not be involved in the usage of S-R-O representations for action selection *per se* but when using them for related processes such as e.g., conflict resolution or performance monitoring during learning.

Interestingly, while we used outcomes that are not intrinsically valuable but assigned motivational relevance to them through instruction, many of the areas we found to be connected to the angular gyrus are also frequently reported in studies using inherently valuable outcomes. This might suggest that their involvement is independent of intrinsic outcome value and fits Daniel and Pollmann (2010) findings that monetary rewards and positive cognitive feedback (correct answer) recruit largely the same brain structures. Also, similar regions were reported in studies on rapid (non-hierarchical) S-R-O learning where outcomes were intrinsically non-incentive as in the present study (Ruge and Wolfensteller, 2013, 2014). On the other hand, we did not find an involvement of the medial frontal areas which are often reported in studies employing rewarding outcomes. In the future, a systematic comparison between the differences and commonalities in processes aiming at intrinsically rewarding vs. abstract outcomes could clarify this issue.

## Conclusion

Together our results support the distinction between representing stimulus-response-outcome contingencies on the one hand and using those representations in the sense of outcome-based response selection on the other hand. Our results suggest that the angular gyrus plays a central role in relating actions in to their sensory outcomes. The actual usage of these representations, such as when explicitly choosing actions according to outcomes, seems to rely on increased functional coupling of the angular gyrus with subcortical as well as prefrontal and cerebellar areas that are engaged in both explicit and implicit processes of action control.

## Conflict of Interest Statement

The authors declare that the research was conducted in the absence of any commercial or financial relationships that could be construed as a potential conflict of interest.
